# Preparation and Characterization of Modified Soda Lignin with Polyethylene Glycol

**DOI:** 10.3390/ma9100822

**Published:** 2016-10-07

**Authors:** Fangda Zhang, Jian Lin, Guangjie Zhao

**Affiliations:** Beijing Key Laboratory of Wood Science and Engineering, Beijing Forestry University, Beijing 100083, China; zfdyale@126.com (F.Z.); zhaows@bjfu.edu.cn (G.Z.)

**Keywords:** soda lignin, polyethylene glycol, melt-spinning, fibers

## Abstract

Soda lignin does not have thermal flowing characteristics and it is impossible for it to be further thermally molded. To achieve the fusibility of soda lignin for fiber preparation by melt-spinning, an effective method for soda lignin modification was conducted by cooking it with polyethylene glycol (PEG) 400 at various ratios. The higher the ratio of PEG that was used, the more PEG molecular chains were grafted at the alpha carbon of the soda lignin through ether bonds, resulting in lower thermal transition temperatures and more excellent fusibility. The modified soda lignin with a weight ratio of lignin to PEG of 1:4 exhibited a relative thermal stability of molten viscosity at selected temperatures. Thereafter, the resultant fusible soda lignin was successfully melt-spun into filaments with an average diameter of 33 ± 5 μm, which is smaller than that of some industrial lignins. Accordingly, it is possible to utilize soda lignin to produce fibrous carbonaceous materials.

## 1. Introduction

As the second most abundant renewable bio-resource, next to cellulose, on the earth, lignin was biosynthesized from the precursor of three monolignols and acted as one of the major cell wall components in higher plants. Although small amounts of lignin in nature were isolated as by-products from pulp and paper manufacturing processes, the amounts of obtained lignin were extremely huge. Generally, around 50 million tons of technical lignins, so-called lignosulfonate, kraft lignin, soda lignin, organosolv lignin, steam explosion lignin, and acid hydrolysis lignin, were produced yearly with various kinds of chemical reagents [1]. However, most technical lignins were burned as fuel to get energy, and only less than 5% of them were utilized as a dispersant in dyestuff and cement, adhesives, and chemicals such as vanillin [2,3,4,5,6]. The existing applications of technical lignins are limited to low value-added products, leading to growing attention being poured into the development of high value-added materials made from lignin.

Lignin is complexly constructed by three monolignols via the main carbon-carbon bonds and ether bonds. It possesses a hexagonal carbon structure and shows a high carbon content of around 60%–65%, implying it is an appropriate precursor for carbonaceous material preparation. Besides, carbon fibers (CFs), as one of the fibrous carbon materials, have excellent mechanical properties and are usually made into composites with thermal setting resin in the applications of aerospace, the military, construction as well as medical and sporting goods [7]. The precursors for commercial CFs are mainly polyacrylonitrile (PAN), while pitch is used for other CF types. However, the explosion of petrochemicals and the high manufacturing cost cause serious environmental pollution and narrow utilization. Therefore, the utilization of technical lignins as raw material for CF preparation seems to be a promising high-value-added process. 

Lignin-based CFs have been successfully prepared by spinning and thermostabilization, followed by carbonization. In the case of spinning, several methods were proposed to produce fibers [8,9,10,11]. Compared with dry spinning and wet spinning as well as electrospinning, melt-spinning does not need solvent for dissolving lignin and can reduce the production cost. However, it requires the good fusibility of lignin when being subjected to heating. Among the technical lignins, Alcell lignin, hardwood acetic acid lignin and hardwood kraft lignin were fusible and could be melt-spun into fibers without any further modification, while the other types of lignins could not be directly converted into filaments [12,13]. These differences in thermal behavior can be explained by not only the frequency of the presence of carbon-carbon condensed structures, such as β-5 and 5-5 linkages of each monolignol in hardwood lignin and softwood lignin, but also the degree of decomposition of the lignin structure during pulping processes [14]. Therefore, several pre-treatments, such as hydrogenolysis [15], phenolysis [16], blending with polymers [12,17,18], organic solvent purification [19,20], and ultrafiltration [21], were conducted to improve the spinnability of partially infusible technical lignins. 

However, there were few investigations on the fiber preparation from hardwood and softwood soda lignin by melt-spinning because of a more condensed structure and poorer thermal fusibility. Accordingly, in this article we describe the efforts directed at the modification and characterization of soda lignin with polyethylene glycol (PEG) 400, and attempt to melt-spin the resultant soda lignin for fiber production. 

## 2. Results

### 2.1. Thermal Analysis of Soda Lignin

**Figure 1:**
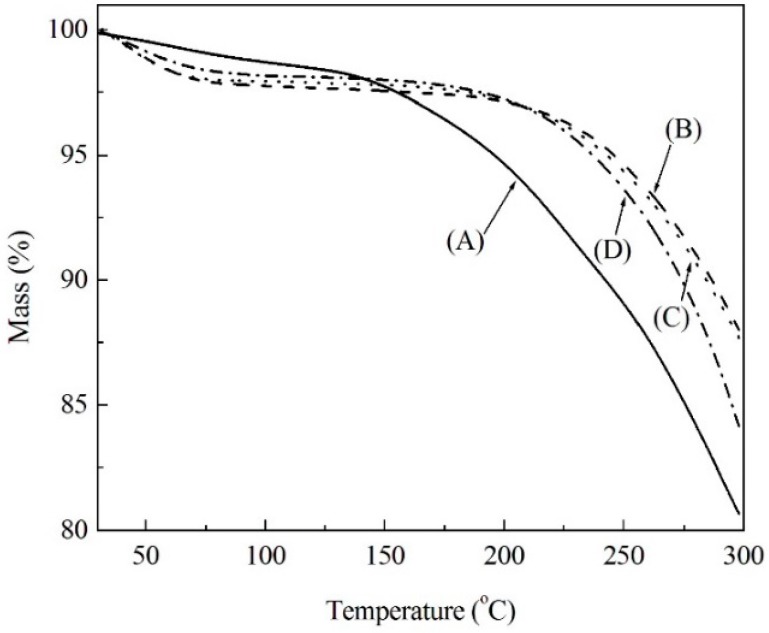
TG curves of soda lignin (**A**) and modified soda lignin with PEG-400 at various weight ratios (**B**) 1:2; (**C**) 1:3; (**D**) 1:4.

Soda lignin powder was isolated from black liquor and subjected to modification with PEG-400 at various weight ratios. The resultant soda lignin exhibited differences in thermal stability by TG measurements as shown in Figure 1. When the temperature was lower than 100 °C, all the weights of the soda lignin slightly decreased because of the loss of adsorbed water molecules, which were derived from the ambient environment. With increasing the temperature, the weight loss gradually increased. Besides, soda lignin showed more weight loss than all modified ones, which may be caused by removing the volatile contaminants in the soda lignin [12,22]. For the modified soda lignin with the weight ratios of 1:2 and 1:3, almost the same thermal behavior was observed, and both of them exhibited less weight loss than that of modified soda lignin with the weight ratio of 1:4. 

**Figure 2:**
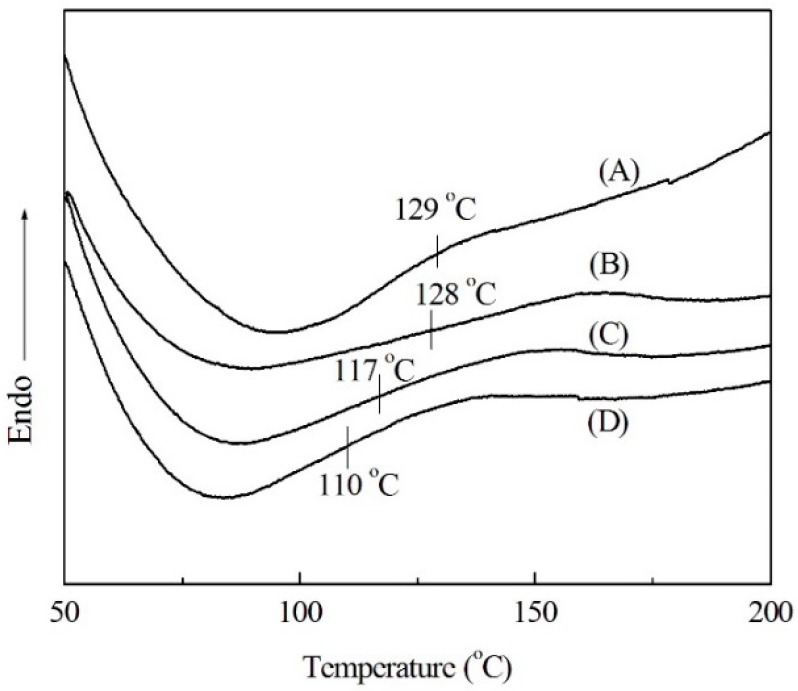
DSC thermograms of soda lignin (**A**) and modified soda lignin with PEG-400 at various weight ratios (**B**) 1:2; (**C**) 1:3; (**D**) 1:4.

Moreover, the high weight loss process was mainly induced by the thermal degradation starting from the decomposition temperature (*T*_d_), which was defined as a temperature that gives a 5% weight loss [13]. The *T*_d_ of modified soda lignin were 267 °C, 262 °C and 253 °C, respectively, which are higher than that of soda lignin at 209 °C. This result was similar to the changes in the glass transition temperature (*T*_g_) of modified soda lignin, which were determined by DSC. As shown in Figure 2, the *T*_g_ of soda lignin gradually declined with increasing the weight ratios of PEG. Besides, the PEG contents in each modified soda lignin prepared with the ratios of 1:2, 1:3 and 1:4 were 19.4%, 24.5% and 31.1%, respectively. Accordingly, the high weight ratio of PEG gave rise to the low *T*_d_ and *T*_g_, which was probably attributed to a greater PEG moiety existing in modified soda lignin prepared with more PEG-400. This was also confirmed by FTIR measurements.

### 2.2. FTIR Spectra Analysis

**Figure 3:**
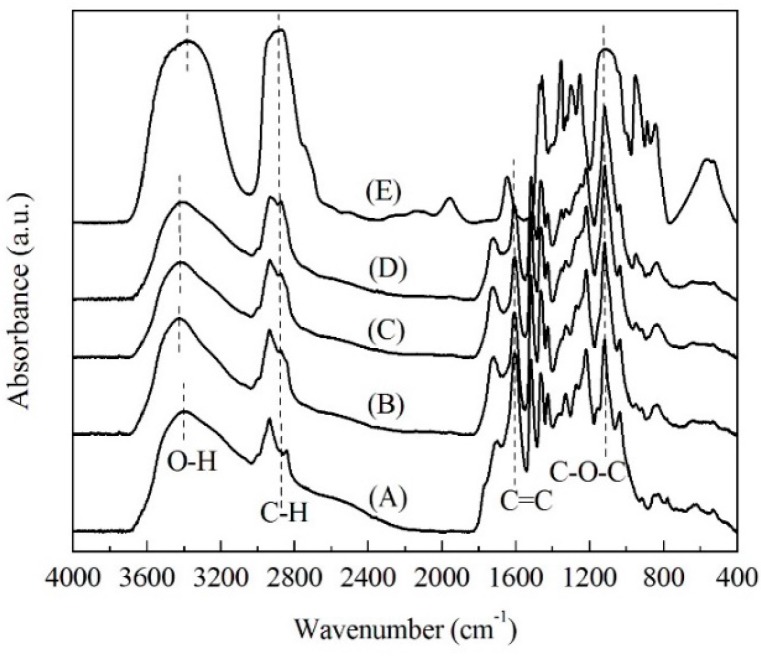
FTIR spectra of soda lignin (**A**) and modified soda lignin with PEG-400 at various weight ratios (**B**) of 1:2; (**C**) 1:3; (**D**) 1:4 and PEG-400 (**E**).

FTIR provides an extremely useful method to investigate functional groups. Figure 3 illustrates the FTIR spectra of soda lignin and modified soda lignin at various weight ratios of PEG. In the spectrum of soda lignin (Figure 3A), the presence of a band centered at around 3397 cm^−1^ was assigned to the O–H stretching vibration of hydroxyl groups [23]. After modification with PEG, the corresponding bands shifted to the higher wavenumbers of 3421 cm^−1^, indicating greater magnitudes of intramolecular hydrogen bonding being formed among the hydroxyl groups in the PEG molecular structures or between lignin and the PEG molecular structures [23,24,25]. It could be clearly proved by the corresponding effects observed in the O–H in the plane bending vibration region (1423 cm^−1^) [23]. 

Further, the relative peak intensity of all modified soda lignin (Figure 3B–D) at 2873 cm^−1^ and 1119 cm^−1^, assigned to the C–H stretching vibration of the methylene groups and the stretching vibration of the C–O groups, respectively, was likely to become stronger than that of the soda lignin without modification with PEG, indicating the greater abundance of methylene groups and ether bonds existing in the modified soda lignin’s molecular structure. Furthermore, as shown in Table 1, the ratio of intensity at 1119 cm^−1^, 2873 cm^−1^ and 3421 cm^−1^ to that at 1600 cm^−1^ (C=C ring stretching vibration), respectively, quantitatively showed the increase with the weight ratio of PEG increasing. Accordingly, the PEG molecular chains were successfully introduced in the lignin structure, and the PEG content increased with the weight ratio of the PEG increasing.

### 2.3. ^13^C-NMR Analysis

**Figure 4:**
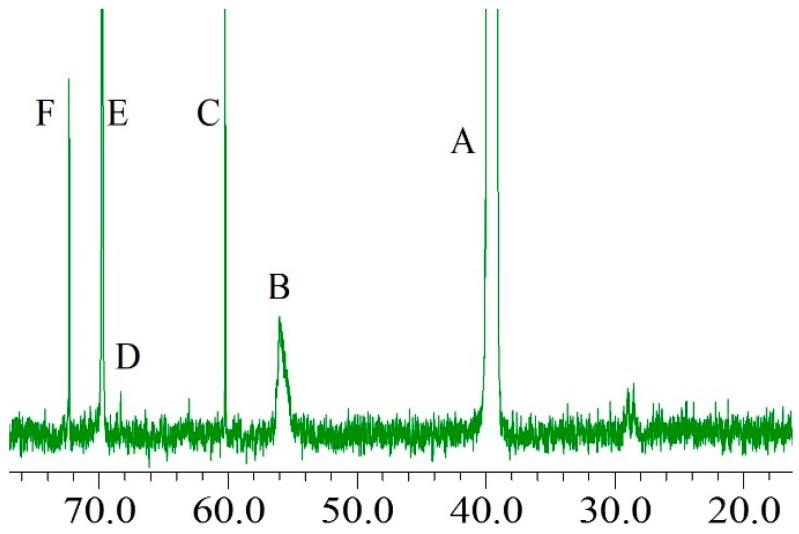
^13^C-NMR spectrum of the modified soda lignin with weight ratio of 1:3.

The PEG moiety was proved to be grafted in the lignin structure, and then the ^13^C-NMR was performed to elucidate the bonded position of the PEG molecule with respect to the lignin. Figure 4 illustrates the ^13^C-NMR spectrum of modified soda lignin with a weight ratio of 1:3. The signals at 39.9 ppm (Figure 4A) and 56.1 ppm (Figure 4B) were assigned to the carbon atom in DMSO-_d6_ of the solvent and to the methoxy groups of soda lignin, respectively, which could be detected at the same chemical shifts in the soda lignin. After modification with PEG, new strong signals at 60.2 ppm and 72.3 ppm (Figure 4C,F) and 69.8 ppm (Figure 4E), which were assigned to the methylene carbon of the alcoholic end groups of PEG and the methylene carbon of the repeating unit of the PEG chain, respectively, were clearly detected [26]. Additionally, a small signal at 68.8 ppm (Figure 4D) was assigned to the methylene carbons bonded to the alpha carbon on the soda lignin side chain through an ether linkage [27,28]. These results strongly suggested that the PEG chain was grafted at the alpha carbon of the soda lignin with an ether bond, which was in good accordance with the FTIR analysis.

In general, the carbohydrate polymers were grafted at the alpha carbon of the lignin through ether bonds in the wood [23]. After the pulping processes with sodium hydroxide, soda lignin was isolated from the carbohydrate polymers. Some hydroxyl groups as reaction sites existed at the alpha carbon position of the soda lignin. When the soda lignin was subjected to heating with PEG-400 under the acidic aqueous condition, the hydrogen cation from the acid was attacked by a pair of electrons donated from the oxygen of the hydroxyl group at the alpha carbon position of the soda lignin. Then the water was simultaneously formed and released from the soda lignin, generating the carbon cation at the alpha carbon position of the soda lignin, which could react with the hydroxyl groups in the PEG chain through the formation of ether bonds. Therefore, the chemical structure of the soda lignin with the PEG bonded at its alpha carbon position could be detected in the 13C-NMR spectrum. 

### 2.4. Viscosity of Soda Lignin and Fiber Preparation

PEG moieties were successfully grafted to the soda lignin structure, probably not only prohibiting the polymerization or condensation of lignin during the heating treatment, but also acting as a solvent and/or plasticizer for the high relative molecular mass fraction [29]. These may give the modified soda lignin high thermal mobility and possibly improve the fusibility. Thereafter, we tried to use the modified soda lignin as a raw material for fiber preparation by melt-spinning. Before processing, the molten viscosity was measured because the correct viscosity of the spinning solution could facilitate continuous spinning and thin fiber production. 

Unexpectedly, it was found that the modified soda lignin with the weight ratio of 1:2 appeared to be partially fusible at the elevated temperatures, and the soda lignin powder could be transformed into viscous molten material. However, only short thick filaments were obtained when picked up with a hairpin, due to the high viscosity. This was confirmed by dynamic viscoelastic measurement. The rotator was difficult to rotate at any shear rates. When increasing the heating temperature to lower the molten viscosity, the decomposition of the modified soda lignin appeared and the release of gases was observed. These indicated that modified soda lignin with the weight ratio of 1:2 was unsuitable for fiber preparation by melt-spinning.

**Figure 5:**
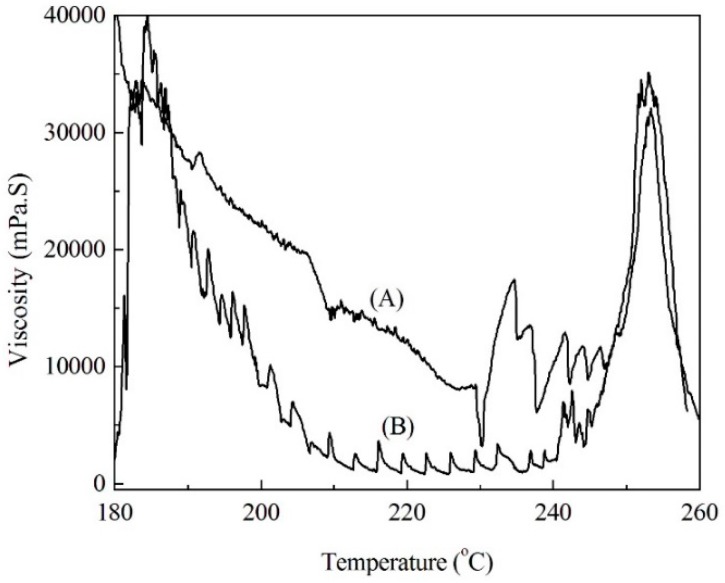
Changes in molten viscosity of modified soda lignin with the weight ratios of 1:3 (**A**) and 1:4 (**B**) with the increase of temperatures.

**Figure 6:**
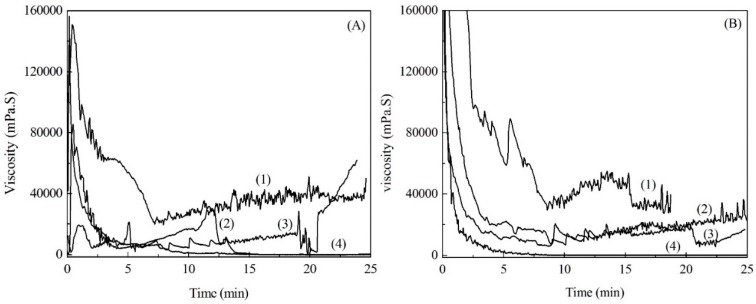
Molten viscosity of modified soda lignin with the weight ratios of 1:3 (**A**) and 1:4 (**B**) at selected temperatures. (Note: (**1**) 180 °C; (**2**) 190 °C; (**3**) 200 °C; and (**4**) 225 °C.)

The changes in the molten viscosity of the modified soda lignins with the weight ratios of 1:3 and 1:4 with the elevated temperatures are shown in Figure 5. The molten viscosity firstly decreased, owing to the movement of the molecular chains of the modified soda lignin by elevated temperatures. Then the decomposition and polymerization of the molten soda lignin resulted in the increase of the molten viscosity as the temperatures increased. Besides, the modified soda lignin with the weight ratio of 1:4 showed a relatively lower molten viscosity than that of the modified soda lignin with the weight ratio of 1:3 during most measured temperatures, which was consistent with the results of the thermal transition temperature measurements. Furthermore, the stability of the molten viscosity at the selected temperatures with a shear rate of 100 1/s was determined, as shown in Figure 6. The molten viscosity of the modified lignin decreased as the shearing time increased. In comparison with the modified soda lignin with the weight ratio of 1:3, the modified soda lignin with the weight ratio of 1:4 exhibited more invariable viscosity at all selected temperatures except for 180 °C. Especially for 225 °C (Figure 6B(4)), almost no change in the viscosity of around 500 mPa·S was observed as the time was prolonged. Accordingly, the modified soda lignin with the weight ratio of 1:4 showed the befitting viscosity for melt-spinning and was subjected to fiber production.

**Figure 7:**
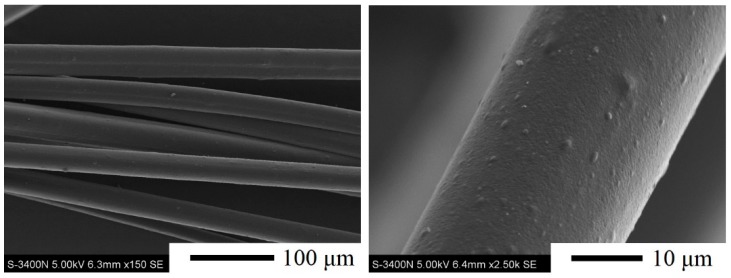
SEM morphology of fibers derived from modified soda lignin with the weight ratio of 1:4.

As expected, the soda lignin–based fibers were successfully prepared from modified soda lignin with the weight ratio of 1:4 by melt-spinning at 225 °C with a winding rate of 56 m/min. The morphology of soda lignin–based fibers is presented in Figure 7. The fibers had a uniform shape, while the surfaces of the fibers were not smooth and the cross-section of a fiber had several pores which may be caused by nitrogen gas for the extrusion of molten lignin under gas pressure. The average diameter of the obtained modified soda lignin–based fibers was around 33 ± 5 μm, which was similar than that of the Alcell lignin–based fibers (31 ± 3 μm) [12], but much smaller than those of softwood and hardwood kraft lignin–based fibers (59 ± 7 μm and 46 ± 8 μm) [12,21], softwood acetic acid lignin–based fibers (84 ± 15 μm) [19] and pyrolytic lignin–based fibers (49 ± 2 μm) [30]. Accordingly, the soda lignin could be considered as a promising raw material for the preparation of fibrous carbon materials.

## 3. Materials and Methods 

### 3.1. Materials

Black liquor powder was obtained from Shanghai Yunzhe new materials Technology Co. Ltd. (Shanghai, China). The content of lignin in black liquor powder is about 30%. Firstly, the powder was homogeneously dissolved in purified water to prepare black liquor. Prior to use, the black liquor was filtrated through filter paper to separate filtrate and residue. After three times filtration, 6 M HCl aqueous solution was slowly added into resultant filtrate with continuous stirring until the pH was around two. The precipitate was then collected by centrifugation at 2270 G for 15 min. Finally, the soda lignin was obtained by freeze drying. Polyethylene glycol (PEG) 400 and sulfuric acid were purchased from SINOPHARM group Chemical Reagent Co., Ltd. (Shanghai, China)

### 3.2. Modification of Soda Lignin

Twenty grams of dried soda lignin was blended with PEG 400 at various weight ratios (soda lignin to PEG-400, 1:2, 1:3 and 1:4) and 95% aqueous sulfuric acid (1.5 wt % on PEG 400) in a round-bottom flask. The flask was immersed in an oil bath that was preheated at 175 °C with continuous mechanical stirring for 2 h. After the reaction, the flask was cooled with cold water and then the cooking product was poured into distilled water with continuous stirring for 30 min Finally, the modified soda lignin was obtained from resultant mixture by centrifugation at 2270 G for 15 min and then freeze drying.

### 3.3. Soda Lignin Melt-Spinning

Fusible soda lignin was subjected to melt-spinning using a single-hole nozzle (diameter: 0.8 mm) at the nozzle temperature of 225 °C and winding rates of 56 m/min under a nitrogen pressure to yield thin filaments.

### 3.4. Characterization

#### 3.4.1. Thermal Analysis

The weight loss behaviors and decomposition temperature (*T*_d_) of soda lignin were measured using TG thermal analyzer (NETZSCH STA 449F3, Selb, Germany) from room temperature to 300 °C with the heating rate of 10 °C /min in high pure nitrogen stream (30 mL/min). Differential scanning calorimetry (DSC) was performed to measure the glass transition temperature (*T*_g_) of soda lignin using the same machine as TG thermal analyzer with a scan rate of 10 °C/min under nitrogen (30 mL/min). Dried soda lignin samples (4 ± 0.5 mg) were placed in a standard aluminum pan with lid and firstly heated from room temperature to 100 °C to expel any remaining moisture in the sample. After cooling, a second DSC trace was then obtained on heating the sample to 300 °C. The glass transition temperature of the samples were recorded as the onset temperatures of the step change in heat capacity in the DSC trace on the second heating process.

#### 3.4.2. Fourier Transform Infrared Spectroscopy (FTIR)

The chemical groups of the soda lignin were examined using FTIR spectrum analysis with GX FT-IR system (PerkinElmer, Norwalk, CA, USA) in the scanning range of 4000 to 400 cm^−1^. The samples were pulverized using size 100 mesh and mixed with potassium bromide at the ratio of 1:100, before being pressed into a disk. 

#### 3.4.3. ^13^C-Nuclear Magnetic Resonance Analysis (^13^C-NMR)

The chemical structure of soda lignin was estimated by NMR analysis. The soda lignin (200 mg) was dissolved in 600 μL of deuterated dimethyl sulfoxide (DMSO-_d6_) as the solvent. The concentration of soda lignin solution was 20 to 30 wt %. The ^13^C-NMR spectrum of the soda lignin solution was recorded using JNM-ECZ400R NMR spectrometer (JEOL, Tokyo, Japan) operating at 150.91 MHz. The spectrum was registered at 25 °C.

#### 3.4.4. Dynamic Viscoelastic Measurement

The molten viscosity of soda lignin was measured using a rheometer with a parallel plate fixture diameter of 20 mm. The distance was set to 1 mm for all of the measurements. The viscosity of soda lignin was determined at the different temperatures and duration of selected temperature with the shear rate of 100 1/s. 

#### 3.4.5. Scanning Electronic Microscopy (SEM)

The surface morphology of soda lignin based fibers were observed using scanning electron microscopy (SEM; S-3400N, Hitachi Co. Ltd., Chiyoda, Tokyo, Japan) at magnifications of 150 to 2000 X and at an accelerating voltage of 5 kV. Before observation, the samples were coated with a thin layer by spraying gold metal using Ion Sputter (E-1010, Hitachi Co. Ltd., Chiyoda, Tokyo, Japan) before searching morphology. The average diameter of fiber was calculated from the individual measurement value of 30 filaments.

## 4. Conclusions

Soda lignin was successfully modified with PEG-400 at various weight ratios. The amount of PEG moieties grafted at the alpha carbon position of the modified soda lignin structure increased as the weight ratio of the PEG used increased, resulting in the low thermal transition temperatures of *T*_d_ and *T*_g_. Modified soda lignin prepared with the weight ratio of 1:4 exhibited a relative thermally stable dynamic viscosity, and it was melt-spun into the soda lignin-based fibers with an average diameter of around 33 μm, which is smaller than those of lignin fibers derived from several types of industrial lignins. Therefore, the modified soda lignin could be considered as a promising raw material for preparing fibrous carbon materials such as carbon fibers and activated carbon fibers.

## Figures and Tables

**Table 1:** FTIR intensity ratios of functional groups in soda lignin (A) and modified soda lignin with PEG-400 at various weight ratios (B) 1:2; (C) 1:3; (D) 1:4.

Intensity Ratio	(A)	(B)	(C)	(D)
*I_C-O_/I_C=C_*	1.08	1.57	1.91	2.03
*I_C-H_/I_C=C_*	0.46	0.69	0.84	1.04
*I_O-H_/I_C=C_*	0.67	0.94	0.95	1.03
